# A remotely-delivered community action project to promote a diabetes lifestyle intervention programme in northwest London: basis, process and outcomes

**DOI:** 10.34172/hpp.2021.30

**Published:** 2021-05-19

**Authors:** Sharan J Kapadia, Yu Gao, Ewa Cumming

**Affiliations:** ^1^Imperial College School of Medicine, London, UK; ^2^Eastbury Surgery, Northwood, London, UK

**Keywords:** Health promotion, Type 2 diabetes mellitus, Health education, Community medicine

## Abstract

**Background** : The prevalence of type 2 diabetes mellitus (T2DM) in London is rising, obesity being a major driver. As part of a primary care placement, the authors (two medical students and a lead general practitioner) directly promoted the Reducing Weight with Intensive Dietary Support (REWIND) programme to patients in Northwest London and collected feedback on the promotion.

**Methods** : The team developed and delivered three remote interventions: a redesigned patient-facing information leaflet, phone calls and text messages, and a live, interactive webinar, to directly engage patients and raise awareness about REWIND. Feedback was collected pre and post-webinar using an anonymised, online survey (essentially functioning as a ‘teaching’ evaluation).

**Results** : Mean interest in REWIND had increased from 2.7 (pre-promotion) to 4.7 (post-promotion), knowledge about REWIND had increased from 2.1 to 4, and self-reported likelihood of enrolling had increased from 2.6 to 4.2 (P<0.01 in all cases). The reported usefulness of the leaflet and webinar was scored 3.7 and 4.4 respectively. Within two weeks of the webinar, two of these patients had joined *REWIND*.

**Conclusion** : Feedback from the patients and GP revealed that the project successfully raised awareness, improved knowledge, and increased the likelihood of enrolment in REWIND. Diabetes programmes and organisations are encouraged to adapt the methods of this project to their own contexts, especially in light of COVID-19 where remote interventions will remain essential.

## Introduction

This community action project on diabetes was a collaboration between two medical students and a lead general practitioner (GP) (authors). In light of COVID-19, it was delivered remotely to a group of eligible patients in Northwood, a region within Northwest London.

In the United Kingdom, one in ten individuals over the age of 40 is diagnosed with type 2 diabetes mellitus (T2DM), costing the National Health Service (NHS) £14 billion annually.^1,2^ Increased diabetes prevalence in recent years is largely attributable to the rising rate of obesity; three in five adults in the United Kingdom are obese.^2^ In London, two in five people are categorised as ‘high risk’ for developing T2DM due to ethnic factors,^1^ and the prevalence of diabetes is 6.65%. In the study area, (Northwood), the prevalence of T2DM is higher than the London average, at 7.75%.^2,3^

The Reducing Weight with Intensive Dietary Support (*REWIND*) programme is a 12-month lifestyle intervention programme for T2DM patients in Northwest London, based on findings from the DiRECT study.^4^ Developed by the United Kingdom Independent Clinical Solutions (UKICS) group, who have previously created other lifestyle programmes such as *Diabetes Prevention*, *REWIND* is implemented in collaboration with local clinical commissioning groups. The programme supports patients to accomplish sustainable weight loss, with the eventual aim of discontinuing medications and perhaps also achieving remission. *REWIND*had recently completed a pilot study with good outcomes and excellent patient feedback.^4^ Given the significantly high prevalence of diabetes in London, as well as the promise that *REWIND*showed, the team was keen to help raise awareness of the programme and increase participation.

To research the programme further and to conduct a needs analysis, the students reviewed the resources on the *REWIND* website and attended a webinar for healthcare professionals. It was discovered that although there were ample resources for healthcare professionals, there could have been more direct promotion and marketing to patients. Given the high quality and affordability of the programme, this stood out as an achievable and high-impact opportunity. The team set out to directly promote *REWIND* to patients with the aim of raising awareness, fostering increased engagement, sparking a conversation around the programme, providing the opportunity for patients to ask questions, and increasing referrals (although patients could not self-refer, the aim was to inform and empower them to request their GP for a referral).

Recruitment strategies for the *PODOSA* diabetes lifestyle trial in 2011^5^ showed that, among South Asians (who comprise a considerable section of the Northwest London population), community-oriented, personalised approaches were far more effective than letters of referral from health professionals. Although *PODOSA*was conducted in a research trial setting, it demonstrated the importance of community outreach for recruitment. Community-centred health promotion is even more important in the current milieu of COVID-19.^6^ Moreover, the importance of technological approaches to health education, especially for chronic diseases, has been well-documented. Whether via webinars,^7^ telephone calls,^8^ or a combination of strategies,^9,10^ significant improvements in quality of life, hospital admission rates, and/or patient-reported satisfaction and knowledge have been reported.^7-10^ Therefore, the students developed three targeted, remote interventions: a redesigned patient-facing leaflet, proactive communication via text messages / phone calls, and a live, interactive webinar held virtually over *Zoom*^®^.

They liaised with the *REWIND* team and the lead GP to seek feedback, better understand the programme’s criteria and logistics, and to explore the needs of the local community. Following their feedback and final approval, the interventions were delivered to eligible patients based on a digital search. During the webinar (final intervention), an anonymous online feedback form was circulated to patients in order to evaluate the project’s impacts.

## Materials and Methods

### Patient search

The students carried out a patient search on the *EMIS Health*^®^ record system using *REWIND*’s inclusion and exclusion criteria. Eligible patients were those with clinical code “Type 2 diabetes mellitus” first diagnosed within the last 12 years, and those on regular DPP4s, SGLT-2s or GLP-1s. No patients were pregnant. Although there was an exclusion filter for patients on insulin (they were not yet eligible, due to the complexity of insulin regimens when dieting), two of them appeared in the final list regardless. This demonstrated a drawback of the patient search system and is explored further under Discussion. The search returned 45 patients.

### Redesigned patient-facing leaflet 

Although the existing patient-facing leaflet was professional and well-designed, the goal was to condense it to a single page for clarity and conciseness, add more graphical elements to improve aesthetics and engagement, include a patient perspective from the pilot study to improve relatability, and include *Zoom*^®^ details for the live webinar. The students followed multimedia design principles such as minimising information to reduce cognitive load and ensuring there was effective ‘alignment’ of text and graphics.^11^ The leaflet provided general information about *REWIND*, including the two pathways (total diet replacement versus low carbohydrate diet) and served as a reminder for the webinar.

**Figure 1:**
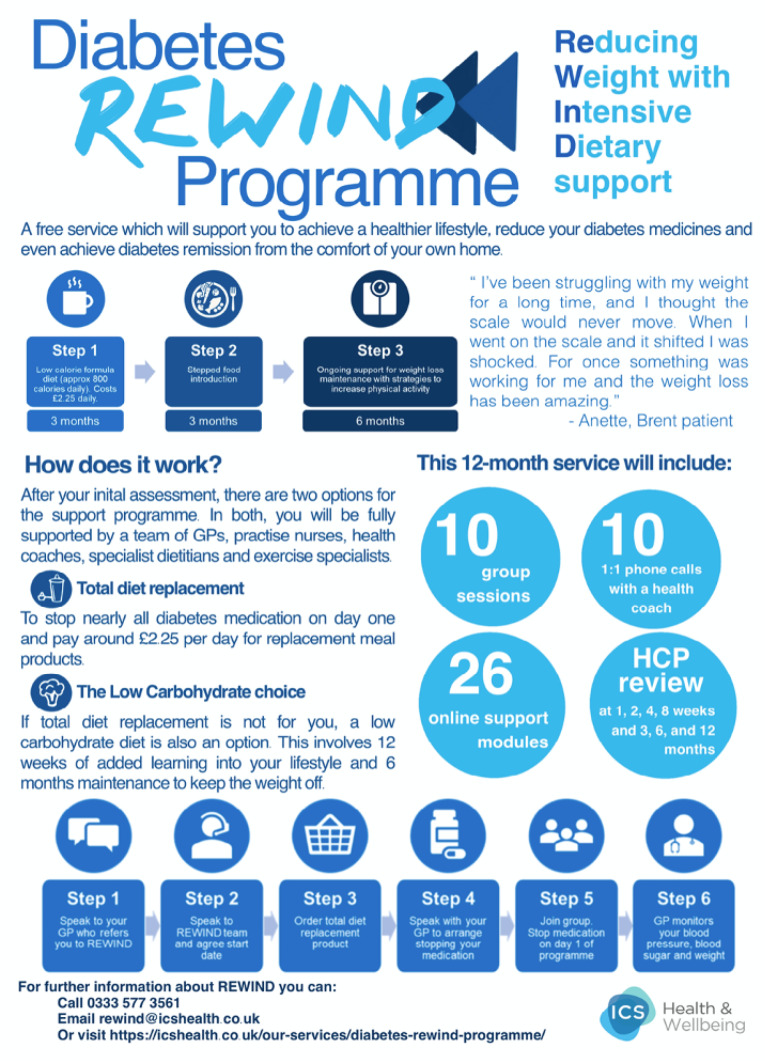
Redesigned patient-facing REWIND leaflet

The redesigned leaflet (Figure 1) was printed in colour (hard copies of the previous *REWIND* leaflet at the GP Surgery were black-and-white) and was posted to the 45 eligible patients. Furthermore, the students uploaded the leaflet to *Google Drive* in full colour and texted patients a digital link via the GP Surgery’s messaging system.

### Text messages and phone calls

To raise awareness of the live webinar and to remind patients about *REWIND*, the students texted and called them. Text messages were sent via the GP Surgery system and contained the link to the *Zoom*^®^ webinar, as well as the aforementioned link to the digital version of the redesigned leaflet. It read:

*“Good afternoon from your GP Surgery. You have been selected as an eligible individual for Diabetes REWIND – a programme for weight-loss, run by ICSUK, to help you stay healthier, slow the progression of your diabetes, and perhaps even stop your medication. We’d encourage you to join our free patient information webinar on Monday 9^th^ November, at 6pm, which includes a Q&A session with the REWIND team: [link to Zoom meeting].”*

A few days before the webinar, the students called a some patients once more as a reminder, to enquire about their initial thoughts on *REWIND*, and to answer some of their preliminary queries. For example, one gentleman asked,

“I am lean and tall – will *REWIND* do much for me?”

The students explained that the programme also covered stress management, exercise, and how to *maintain* weight loss. He was encouraged to attend the webinar in order to receive first-hand information and an opportunity to pose questions directly to the *REWIND*team. Encouragingly, he did.

### Interactive live webinar 

The live webinar was held on Monday, 9^th^ November 2020 at 6 pm. Lasting two hours, it was attended by 11 of the 45 patients. Aside from the three authors, the panel of hosts comprised three members of the *REWIND*team, including the Diabetes Commissioner for the region, as well as a patient representative who had recently enrolled into the programme.

Helmink^12^ discussed that effective promotion of a lifestyle programme ought to facilitate an active patient role, convince patients that the intervention is “effective and safe”, and foster motivation, self-efficacy, and enjoyment. According to self-determination theory, *intrinsic* motivation is most likely to lead to sustained adherence and improved health behaviours. Promotion should also reinforce links between lifestyle improvement and clinical improvement. Moreover, stigma and isolation are common among people with diabetes,^13^ a phenomenon which has no doubt been exacerbated by COVID-19. The students were therefore keen to provide a group setting that would offer patients a reprieve.

With the above in mind, the students ensured the webinar was interactive and engaging, both in content (concise and colourful) and delivery (positive and action-oriented), in order to keep the session patient-centred. The information was kept up-to-date and reliable by inviting the *REWIND* team, and patients’ intrinsic motivation was boosted by inviting the patient representative to share his experiences and address any doubts. The webinar was structured as follows:

- Introductions and acknowledgements
- Overview of *REWIND*, including the aims, the dietary pathways, ongoing support, a discussion on “What makes losing weight difficult for you?”, results from the pilot study, and a graphical timeline from the *Know Diabetes* website.
- Detailed exploration of the two pathways, including costs and meal choices.
- Review of eligibility criteria and the referral process.
- Distribution of a feedback form for project evaluation. The form link was displayed at this point and the form could be filled in any time until the end of the webinar.
- Perspectives from the patient representative, including challenges, motivators and advice.
- Comprehensive, 45-minute Q&A session by the REWIND panel and patient representative.
- Signposting to further information and a reminder to complete the feedback form.

To ensure the webinar was engaging, the students sought to keep their narration well-aligned with the slides to improve temporal contiguity. Redundancy was minimised and “segmenting” techniques were used to keep slides simple.^11^ In order to ensure engagement and recall, effective non-verbal communication and a lively tone were maintained.^14^

## Results

Respondents were asked to score their interest in the programme, knowledge about the programme and self-reported likelihood of enrolling out of 5. Scores were requested for “3 weeks ago” versus “now” (post-intervention). Two-tailed, paired t-tests were used to compare pre- and post-intervention scores. Next, respondents were asked to score the ‘usefulness’ of the webinar and the leaflet out of 5. Finally, there was a free-text box for further comments. A spreadsheet was used to analyse the data. 11 responses were received, though one was excluded as the free text suggested it was from a member of the *REWIND* team (thus n = 10).

The results were promising. The mean interest in *REWIND* had increased from 2.7 to 4.7, knowledge about *REWIND* had increased from 2.1 to 4, and self-reported likelihood of enrolling had increased from 2.6 to 4.2 (*P* < 0.01 in all cases). The reported usefulness of the leaflet and webinar was scored 3.7 and 4.4 respectively. Within two weeks of the webinar, two of these patients already joined *REWIND*, with further referrals expected.

## Discussion

The results, although derived from a small sample, show that the project produced a significant increase in awareness, knowledge and likelihood of joining.

### Enablers 

An important reason for the success of this project was the students’ close collaboration with the *REWIND* team, the GP surgery and the community – this directly engaged community members and fostered direct and personalised discussion aboutthe programme. Moreover, the team’s community-focussed outlook increased this project’s reach and fostered confidence that they were on the ‘right track’, serving a real need.^5,6^ The students regularly sought feedback to ensure the content and design were appropriate and kept the channel of communication open via e-mail and social media.

The webinar received excellent feedback. Although it should be noted that scores for ‘interest’, ‘knowledge’ and ‘likelihood of joining’ included patients who had not heard of the programme before, the results are nevertheless encouraging. It is likely that the direct engagement with patients was key. The webinar enabled focus-group style discussion about *REWIND* in a safe and open space. Careful planning also helped the webinar run smoothly – indeed, one patient commented that the project was “well organised”. The presence of the *REWIND* team in the webinar was highly valuable – it bolstered the webinar’s credibility and ensured that patients’ concerns and queries (for example, “how is this different to a fad diet?”) were addressed with accurate, reliable and up-to-date information in the comprehensive Q&A session. In addition to providing a much-needed perspective, the patient representative was able to relate to the patients, address their apprehensions and potential barriers to participation, boost their motivation by sharing personal stories, and help answer queries such as “is this programme for me if I am lean”. Indeed, it has been reported that involving community members directly to learn more about the potential barriers to participation, such as beliefs and attitudes about diabetes, can improve recruitment.^15^ Moreover, the *REACH* programme^13^ highlighted that involving community health workers is valuable for health promotion. In a way, the patient representative, being from Northwest London himself, acted as a community health worker; he related and responded to cultural and community norms to enable acceptance.^16^

### Challenges

Despite adding ‘insulin’ as an exclusion criterion, two patients on insulin appeared in the final search regardless (perhaps due to poor coding). Unfortunately, a limitation of the GP Surgery’s software was the inability to exclude individual patients from a search manually. Re-running the filters did not help. The students proceeded in the interest of time; these patients received a webinar invite along with the other 43. When they joined the webinar, the students apologised and explained that although patients on insulin are not currently eligible, there were plans to expand the inclusion criteria soon and encouraged them to remain logged in. It was heartening to know that not only did they decide to stay until the end, they fed back that it was highly valuable. Nevertheless, this demonstrates that patient filters are not infallible; refinements in patient search technology, such as the ability to exclude individual patients manually prior to sending out a mass message, may be beneficial. Including a brief reminder of eligibility criteria in the text message may also have helped.

The leaflet was well-received by the *REWIND* team, who have since requested a copy for further promotion. As discussed below in Sustainability, this leaflet will also be uploaded to the GP Surgery’s website. One potential improvement that inadvertently remained undetected, was the use of the word “free” in the leaflet. Technically, although the support services such as the online modules, health coaching and workshops are without cost, patients on the ‘Total Diet Replacement’ pathway do need to pay for special meal powders. In practice, this often *saves* patients money because their spending on groceries significantly decreases. Nevertheless, the leaflet could have been more explicit.

Conducting a health promotion session remotely was not without its challenges. Many of the key attributes of effective small-group teaching sessions are associated with the physical environment, such as eye contact and being in the same room.^17^ The element of connection is more challenging to achieve online, where the distance feels vast, and true eye contact is non-existent. Thus, running the webinar online required careful thought on maximising engagement. The team sought to achieve this by ensuring attendees kept their video on, keeping the tone relaxed, and by integrating interactivity throughout by encouraging attendees to speak aloud as well type in the chat box. It is perhaps worth noting, however, that the remote delivery of the interventions may have fit better around patients’ work schedules – they were able to join the webinar from any internet-enabled location. This convenience could also explain the patients’ high level of interest in *REWIND* following the project – since *REWIND*will be remotely delivered this year, patients may have felt that it could fit well around their schedules. Indeed, during an analysis of barriers to participation in a lifestyle intervention for cancer survivors,^18^ “inconvenience to everyday life” and “transport to trial site” featured in over 60% of responses. Moreover, the patients may have enjoyed the remote style, as suggested by feedback from two patients (“well organised”) and may be looking forward to the remote sessions within *REWIND*.

Another challenge was technological accessibility; almost all patients invited were over the age of 60 and technology literacy in the elderly remains limited.^19,20^ Indeed, when the students called patients prior to the webinar, some expressed uncertainty regarding their ability to use platforms such as *Zoom*^®^. This accessibility issue may have contributed to the relatively low conversion rate from invited patients to those who attended the webinar. Moreover, there may have been some patients who neither received the printed leaflet in the post, nor were able to open the digital version on their mobile phone. In future, perhaps phone-based pre-webinar workshops for patients, explaining how to join virtual meetings and access digital documents, would be beneficial.

The team worked on a tight timeframe during a relatively short clinical placement. Given more time, the students could have sent the leaflet and text messages earlier, providing a longer interval before the webinar. This may have increased attendance. Had their clinical placement not ended soon after, the students could have run more webinars for patients who could not attend the first. Although the attendance was sufficient to run a rich, interactive and engaging webinar, it was a relatively small sample size for feedback purposes.

### Sustainability

The webinar was recorded with consent from all attendees. The *REWIND*team plans to upload it online, including on their website. A version of the leaflet without the webinar date and Zoom(R) link will be uploaded to the Surgery’s website. Furthermore, since the students actively involved the GPs and the Practice Manager throughout; they will be able to actively promote *REWIND* to patients in consultations. This will further increase the likelihood that patients are signposted to the project resources. Finally, the project has led the GP surgery to further recognise and appreciate the value of using modern technology to promote a range of interventions such as cancer screening programmes, childhood immunisation, and other lifestyle programmes.

The study had a small sample size; more attendees would have led to more survey responses, which may have improved the reliability of results. Given more time, perhaps more intensive marketing could have been conducted, or multiple webinars held.

The anonymous survey contained predominantly quantitative questions which were analysed statistically to limit bias. Bias could have been further reduced by involving an independent data analyst (although this was not feasible given the scale and time constraints).

### Reflection

Although small-scale, the team believes the work was impactful, and that it will continue to benefit the community in the long-term. The students found the project personally developmental and rewarding; they learned about the processes and challenges of designing and delivering a community initiative and honed their communication skills. Crucially, they gained a first-hand understanding of the psychosocial determinants of health-seeking behaviour, such as anxieties, cost, and language barriers.

## Conclusion

Overall, remote promotion of the *REWIND* programme directly to patients successfully raised awareness, enhanced knowledge, and increased likelihood of enrolment. The webinar approach fostered direct engagement and was highly valuable. Diabetes programmes and organisations are encouraged to adapt the methods of this project to their own contexts, especially in light of COVID-19.

## Acknowledgements

The authors would like to sincerely thank all patients involved, the *REWIND* team, patient representative Denis Cullen, GP Dr Amanda Shanmugarajah, Practice Manager Ms Rupal Nathwani, and the reception staff at Eastbury GP Surgery for their support.

## Funding

Nil.

## Competing interests

None.

## Ethical approval

Not applicable.

## Authors’ contributions

SJK, YG and EC were all involved in design, conceptualisation and implementation of the project. SJK (corresponding author) carried out data analysis, statistical analysis and interpretation, and wrote the manuscript. YG and EC helped with the manuscript. EC supervised the project.
